# Improved Intestinal Permeation of Cyclosporin a by FCIGRL-Modified Tight Junction Modulator in Rats

**DOI:** 10.3390/pharmaceutics17111395

**Published:** 2025-10-28

**Authors:** Dong-Ho Jeong, Jung-Woo Kim, Keon-Hyoung Song

**Affiliations:** 1 Department of Pharmaceutical Engineering, College of Medical Sciences, Soonchunhyang University, Asan 31538, Republic of Korea; 2 R&D Center, Jinyang Pharm. Co., Ltd., Seoul 08826, Republic of Korea

**Keywords:** permeation enhancer, tight junction modulator, zonula occludens toxin, cyclosporin A

## Abstract

**Objectives:** Cyclosporin A (CsA) is an immunosuppressive drug that is highly effective. CsA, similar to other drugs with limited oral bioavailability due to poor membrane permeability, requires the use of absorption enhancers in its formulations. Phe-Cys-Ile-Gly-Arg-Leu (FCIGRL-OH), a peptide fragment of Zonula occludens toxin (ZOT), has been studied for its potential to enhance drug absorption by regulating intercellular tight junctions. This study aimed to evaluate the effects of four novel modified peptides, which have been substituted or dimerized at the C-terminus or cysteine moiety of FCIGRL-OH, as improved versions of FCIGRL-OH on the intestinal permeation of CsA. **Methods:** The four modified peptides used were FCIGRL-NH_2_ (Pep-1), homo-dimer peptides derived from FCIGRL-OH and Pep-1 (Pep-2, Pep-3), and a peptide in which the cysteine in Pep-1 was replaced with N_3_-substituted dipropionic acid (Pep-4). Pharmacokinetic analysis was performed following intraduodenal administration of CsA with each of four peptides in the presence of levan and benzalkonium chloride (BC) in rats. **Results:** Results showed that each of Pep-2, Pep-3, and Pep-4 significantly increased intestinal absorption of CsA in the presence of levan and BC. In particular, the area under the curve (AUC_0–360min_) for CsA was significantly enhanced by 2.01-fold (*p* < 0.01) and 2.03-fold (*p* < 0.05) when treated with Pep-3 and Pep-4, respectively, at a dose of 10 mg·kg^−1^. Additionally, the maximum plasma concentration (C_max_) of CsA increased by 2.46-fold (*p* < 0.01) with Pep-3 and by 2.37-fold (*p* < 0.01) with Pep-4. **Conclusions:** These study findings indicate that Pep-2, particularly Pep-3 and Pep-4, are involved in tight junction opening as novel absorption enhancers for intestinal delivery of CsA.

## 1. Introduction

Cyclosporin A (CsA) is a potent immunosuppressive drug used to treat autoimmune diseases and prevent organ rejection [1]. Like many other drugs that have limited oral bioavailability, CsA has a poor membrane permeability due to its high molecular weight and rigid structure of cyclic 11 amino acids [2,3,4]. One approach to improving oral delivery of CsA is using permeation enhancers that can increase drug absorption across the intestinal membrane [5,6].

Zonular occludens toxin (ZOT) is an endotoxin produced by *Vibrio cholerae*. It is a single polypeptide chain of 399 amino acids that has been evaluated as a new class of tight junction-modulating permeation enhancer for drug delivery [7,8,9,10]. ZOT has been shown to increase mucosal permeability without causing tissue damage. It can also enhance the absorption of varying molecular weight markers (PEG4000, mannitol, sucrose, and inulin) and therapeutic agents (CsA, doxorubicin, acyclovir, and paclitaxel) across Caco-2 cell monolayers [10,11,12]. Ongoing size truncation studies of ZOT have also shown that FCIGRL-OH, 288-293 amino acid residues of ZOT, retains the ZOT domain directly involved in the tight junction modulation [13,14,15,16]. FCIGRL-OH improves the intranasal absorption of paracellular markers and low bioavailable agents such as PEG4000, inulin, mannitol, calcitonin, saquinavir, and ritonavir in a non-toxic manner in rat studies [13,14,15,16]. Additionally, FCIGRL-OH significantly increases AUC and C_max_ of CsA following intestinal administration with protease inhibitors (PIs) and benzalkonium chloride (BC) in rats [17].

Recent studies have shown that a C-terminal amidated peptide derived from FCIGRL-OH can lead to enhanced drug absorption when administered with mannitol and atenolol [18,19]. Additionally, a structure-activity relationship study has demonstrated that modifying the cysteine residue at position 2 of FCIGRL-OH can generate more potent analogs [20]. These findings on amino acid modifications of FCIGRL-OH suggest that further stabilization of the peptide by altering chemically reactive sites, such as the cysteine and the C-terminus of FCIGRL, could enhance its permeation-enhancing properties. This, in turn, could improve the intestinal permeability of CsA. Consequently, this study prepared four modified peptides in which the cysteine at position 2 of FCIGRL was either dimerized or replaced with an N_3_-substituted dipropionic acid, while the C-terminus of FCIGRL was capped with an amide, which were selected based on preliminary experiments.

The study aimed to evaluate the efficacy of these four novel peptides as absorption enhancers and assess their impact on the intestinal permeation of CsA. To focus on the effectiveness of the amino acid modifications and minimize potential variability in the effects of the four peptides, each CsA solution was prepared with levan, a fructose polymer, and BC, which are known to provide additional intestinal metabolic protection to peptides [17,19,21,22]. Moreover, each CsA solution was administered directly into the duodenum of rats, as the peptides are likely to undergo significant gastric degradation in the stomach. Pharmacokinetic analysis was performed after intraduodenal administration of CsA with each FCIGRL-modified peptide in the presence of levan and BC in rats.

## 2. Materials and Methods

### 2.1. Materials

Four peptides with a purity of 95% were purchased from AnyGen Co., Ltd. (Gwangju, Republic of Korea), synthesized using the Fmoc solid-phase peptide synthesis method. AnyGen Co., Ltd. provided a certificate of analysis for each peptide. These peptides, which were modified or dimerized at the C-terminus or the cysteine residue of the 288-293 amino acid fraction of ZOT, were stored at −70 °C prior to evaluating their effects on the intestinal permeation of cyclosporin A (CsA). Levan, used as one stabilizer for the peptides, was generously donated by RealBioTech Co., Ltd. (Gongju, Republic of Korea). The following high-purity reagents were obtained from Sigma Chemical Co. (St. Louis, MO, USA): CsA, benzalkonium chloride (BC) as the other stabilizer for the peptides, cyclosporin D as an internal standard for high-performance liquid chromatography (HPLC) analysis, formic acid (≥95%), and ammonium formate (97%) as additives of mobile phases. Polyethylene tubing (outer diameter of 0.965 mm; PE-50) and surgical supplies used in animal experiments were acquired from Becton Dickinson (Sparks, MD, USA) and Professional Hospital Furnishers (Punjab, Pakistan), respectively. Zoletil 50, an anesthetic, was obtained from Virbac (Carros, France) with the approval of the Ministry of Food and Drug Safety of Korea. HPLC-grade Methanol, used as a mobile phase for analysis, and HPLC-grade ethanol, used for preparing solutions, were acquired from Fisher Scientific (Fair Lawn, NJ, USA). All solutions used for analysis and solution preparation were purified using an Arium Mini Plus system (H20-MA-T, Sartorius, Göttingen, Germany) and subsequently filtered through cellulose nitrate membrane filters (0.2 µm, 47 mm, Whatman, Maidstone, UK).

### 2.2. Animals

Male Sprague–Dawley rats, each weighing between 280 and 290 g, were individually housed and given standard rat chow while being maintained on a 12-h light/dark cycle for a minimum of 2 days after arriving from Koatech Corp. in Pyeongtaek, Republic of Korea. The rats were fasted overnight prior to the administration of any treatments, although they had free access to water. The animal study protocol was approved by the Institutional Animal Care and Use Committee of Soonchunhyang University under reference number SCH21-0007.

### 2.3. Preparation of CsA Solutions for the Animal Study

Intraduodenal solutions containing CsA and modified peptides were prepared for administration to rats. Each solution included one of four modified peptides, along with levan, BC, and ethanol. The control group consisted of a solution that contained CsA, levan, BC, and ethanol, but lacked the peptides to assess the effect of each individual peptide. Levan and BC served as adjuvants to stabilize the peptides, while ethanol acted as a solubilizer for the components. Based on preliminary studies, the final concentrations of each component in the solutions were carefully determined and prepared immediately prior to administration to the rats. The concentrations used were as follows: CsA at 2 mg·mL^−1^, each peptide at either 5 mg·mL^−1^ or 2.5 mg·mL^−1^, levan at 0.5 *w*/*v*%, BC at 0.5 *w*/*v*%, and ethanol at 5 *v*/*v*%.

### 2.4. Intraduodenal Administration of CsA Solutions to Rats

Rats were administered each CsA solution through the duodenum, and the temporal plasma concentration profiles were monitored over a 360-min period. Anesthesia was induced with an intramuscular injection of Zoletil 50 at a dosage of 25 mg·kg^−1^. The femoral artery and vein were cannulated using PE-50 tubing. After cannulation, each solution was administered at a volume of 2 mL·kg^−1^ slowly to the duodenum. Blood samples of 250 µL were collected into polypropylene tubes at the following time points: 20, 40, 60, 120, 180, 240, 300, and 360 min after administration. These samples were immediately centrifuged to obtain plasma samples of 100 µL. The deproteinization process for analysis involved adding 500 µL of methanol with the internal standard to each tube and vortexing for 30 s. The samples were centrifuged again at 18,000× *g* for 15 min at 4 °C again. The supernatants were analyzed using an HPLC-Mass spectrometer system.

### 2.5. HPLC-Mass Spectrometer Conditions

Quantitative analysis of CsA was performed using an HPLC-Mass spectrometer system (Shimadzu, Kyoto, Japan), which included a degasser (DGU-20A), a binary pump (LC-20AD), an autosampler (SIL-20A), and a mass spectrometer (MS-2020). After the deproteinization process, a 10 µL plasma sample of CsA was injected into a C_18_ reversed-phase column (Vydac, 2.1 × 150 mm, 5 μm particle size; Hesperia, CA, USA). The plasma samples were separated through the column using gradient conditions at a flow rate of 0.2 mL·min^−1^. The mobile phase consisted of two eluents: Eluent A was a 10 mM aqueous solution of ammonium formate, which contained 0.1% formic acid, while Eluent B was methanol also containing 0.1% formic acid. The gradient conditions included increasing Eluent B from 55% to 100% over 2 min, maintaining this composition for 2 min, then decreasing back to 55% over 1.5 min, and finally maintaining this for 7 min. The mass spectrometer was equipped with an electrospray ionization source and was operated in positive-ion, selected-ion monitoring mode at *m*/*z* 1203.0 of a protonated molecule [M+H]^+^. The desolvation line temperature was set to 250 °C, the heat block temperature to 200 °C, the nebulizing gas flow rate to 1.5 L·min^−1^, and the drying gas flow rate to 15 L·min^−1^. High-purity nitrogen (>99.999%) was utilized as both the nebulizing and drying gas.

### 2.6. Data Analysis

The plasma concentration of CsA was assessed by converting the peak area of CsA in the chromatogram of selected-ion monitoring mode obtained from HPLC-mass spectrometry into concentration values. This conversion was conducted using the ratio of the peak area of cyclosporin D as the internal standard. The calibration curves of CsA in plasma demonstrated good linearity, with squared correlation coefficients (*r*^2^) of 0.9999. The limit of quantification was determined to be 20 ng/mL. Pharmacokinetic parameters, such as maximum plasma concentration (C_max_), the area under the plasma drug concentration-time curve (AUC_0–t_), half-life (T_1/2_), clearance (Cl), time to reach the maximum concentration (T_max_), and volume of distribution (V_d_), were calculated over time using non-compartmental analysis with WinNonlin^®^ software (version 5.3; Pharsight, Mountain View, CA, USA). The enhancement ratio for each pharmacokinetic parameter was calculated as the ratio of each parameter in the control group. All results were presented as mean ± standard error of the mean (mean ± SEM). Statistical significance was analyzed using SPSS for Windows version 12.0 (SPSS Inc., Chicago, IL, USA), employing Student’s *t*-test and one-way analysis of variance to evaluate significance levels at *p* < 0.01 or *p* < 0.05.

## 3. Results

### 3.1. Structures of FCIGRL-Modified Peptides

Four novel peptides have been developed as improved versions of FCIGRL-OH. These peptides have undergone substitutions or dimerization at the C-terminus or the cysteine moiety of FCIGRL. Importantly, they retain the ‘-IGRL-’ amino acid sequence, suggesting they are likely to regulate intercellular tight junctions similarly to ZOT or FCIGRL. Pep-1 was a C-terminal modified version of FCIGRL-OH, where an amino group replaced the hydroxyl group. Pep-2 and Pep-3 were homo-dimer peptides derived from FCIGRL and Pep-1, respectively. Pep-4 was another modified version of Pep-1, where the cysteine has been replaced with N_3_-substituted dipropionic acid (Table 1).

### 3.2. Intraduodenal Administration of CsA with FCIGRL-NH_2_, C-Terminal Amidated FCIGRL

**Figure 1:**
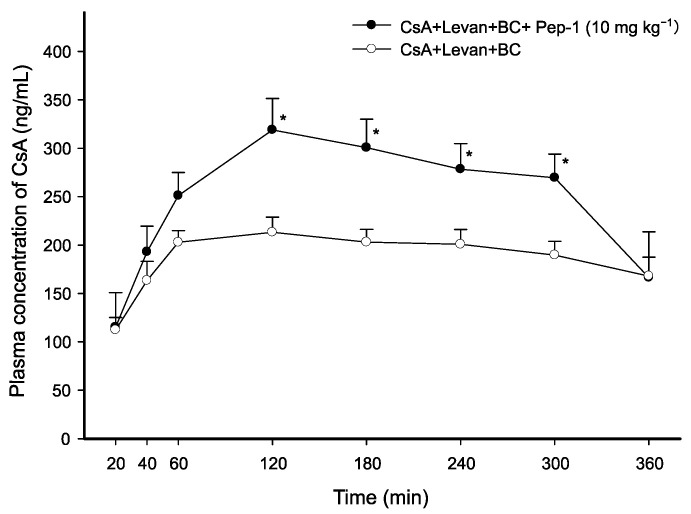
Average plasma concentration of CsA over time following intraduodenal administration of each CsA solution with Pep-1, levan, and BC. Each data point represents the mean ± standard error of the mean (SEM) of 3–4 rats. 2 mg·kg^−1^ of CsA and 10 mg·kg^−1^ of Pep-1 in levan (0.5 *w*/*v*%) and BC (0.5 *w*/*v*%) solution were prepared. Significant differences are indicated with * *p* < 0.05 when compared with CsA/levan/BC control solution at each time point.

Male Sprague-Dawley rats were used to confirm the efficacy of Pep-1 as an absorption enhancer for CsA. After cannulation in both the femoral artery and vein, they were randomly administered a control solution of CsA and solutions containing CsA along with peptides. All solutions included levan, BC, and ethanol at the following concentrations: CsA at 2 mg·mL^−1^, 10 mg·kg^−1^ of Pep-1, levan at 0.5 *w*/*v*%, BC at 0.5 *w*/*v*%, and ethanol at 5 *v*/*v*%. Figure 1 illustrates the mean plasma concentrations (±SEM) of CsA over time following the intraduodenal administration of CsA solutions. Results indicated that the Pep-1 peptide in the CsA/levan/BC solution increased plasma levels of CsA by 1.50-fold (*p* < 0.05), 1.48-fold (*p* < 0.05), 1.39-fold (*p* < 0.05), and 1.42-fold (*p* < 0.05) at 120 min, 180 min, 240 min, and 300 min, respectively, compared to the control (CsA/levan/BC solution alone).

Pharmacokinetic parameters calculated after administering CsA showed that Pep-1 including levan and BC increased the C_max_ of CsA by 1.50-fold (*p* < 0.05). However, there were no statistical differences in AUC_0–360min_ between the two groups, although Pep-1 increased the AUC_0–360min_ by 1.36-fold (Table 2). These results indicate that 10 mg·kg^−1^ of Pep-1 did not cause a significant difference in the absorption extent of CsA intraduodenally in the presence of levan and BC.

### 3.3. Effect of FCIGRL Homo-Dimers on Intraduodenal Absorption of CsA

To determine whether cysteine-dimerization could affect the permeation-enhancing effect of FCIGRL and evaluate intraduodenal absorption of CsA in the presence of levan and BC, four different solutions were prepared: (1) CsA/levan/BC as control (2 mg·kg^−1^ of CsA, levan (0.5 *w*/*v*%), and BC (0.5 *w*/*v*%)), (2) CsA/levan/BC with 10 mg·kg^−1^ of Pep-2, (3) CsA/levan/BC with 5 mg·kg^−1^ of Pep-3, and (4) CsA/levan/BC with 10 mg·kg^−1^ of Pep-3.

**Figure 2:**
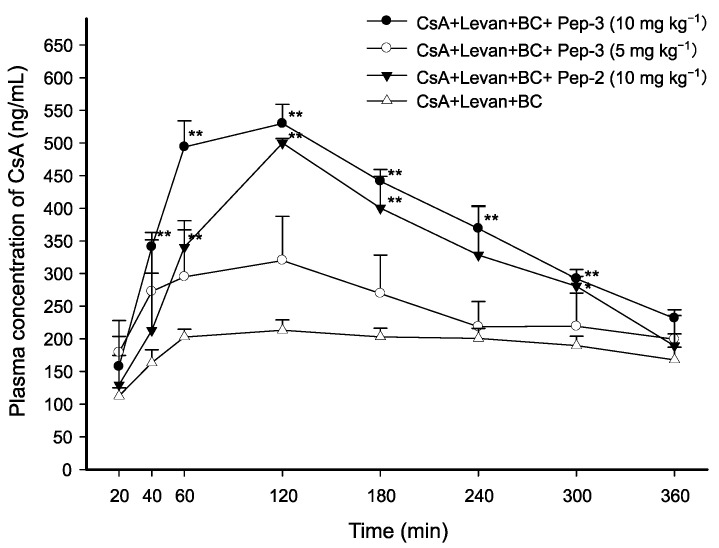
Average plasma concentration-time profiles of CsA after intraduodenal administration of CsA solutions with varying concentrations of Pep-2 and Pep-3. The dose of CsA was 2 mg·kg^−1^. Data are presented as mean ± SEM of 3–4 rats. Significant differences are indicated with * *p* < 0.05 and ** *p* < 0.01 compared with CsA/levan/BC control solution.

As shown in Figure 2, the CsA solution containing levan, BC, and 5 mg·kg^−1^ of Pep-3 did not result in a statistically significant increase in plasma concentrations of CsA compared to the control solution of CsA/levan/BC at any time points. Additionally, there was no significant difference in the AUC_0–360min_ or C_max_ of CsA between the two groups (Table 2). This indicated that the absorption of CsA was not affected by 5 mg·kg^−1^ of Pep-3. However, when the concentration of Pep-3 was doubled to 10 mg·kg^−1^, there were significantly increases in the AUC_0–360min_, which rose by 2.01-fold (*p* < 0.01) and, in the C_max_, which increased by 2.46-fold (*p* < 0.01). This enhancement resulted in elevated plasma concentrations of CsA across all time points from 40 to 300 min. Specifically, the increases were 2.09-fold at 40 min, 2.44-fold at 60 min, 2.48-fold at 120 min, 2.17-fold at 180 min, 1.84-fold at 240 min, and 1.54-fold at 300 min (*p* < 0.01).

Another significant observation was the impact of Pep-2 on the absorption of CsA. Pep-2-containing CsA/levan/BC solution at 10 mg·kg^−1^ increased CsA concentration in plasma by 1.68-fold (*p* < 0.01), 2.35-fold (*p* < 0.01), 1.97-fold (*p* < 0.01), and 1.48-fold (*p* < 0.05) at 60, 120, 180, and 300 min, respectively, compared to the control (Figure 2). Moreover, the Pep-2 solution significantly increased AUC_0–360min_ and C_max_ by 1.74-fold (*p* < 0.01) and 2.32-fold (*p* < 0.01), respectively, compared to the control (Table 2).

These results indicate that cysteine dimerization and the concentration of peptide can significantly affect intraduodenal absorption of CsA, suggesting that 10 mg·kg^−1^ of Pep-2 or Pep-3 is required for further enhancement of CsA absorption. Additionally, when administered at the same dose of 10 mg·kg^−1^, the Pep-3 solution had higher AUC_0–360min_ and C_max_ of CsA than the Pep-2 solution, indicating that C-terminal amidation could increase the intrinsic permeation-enhancing property of the peptide.

### 3.4. Effect of Cysteine-Substituted FCIGRL on Intraduodenal Absorption of CsA

To evaluate the enhancing effect and the impact of concentrations of Pep-4 on the absorption of CsA, CsA solutions of two different concentrations of Pep-4, a peptide that replaced a cysteine residue of Pep-1 with N_3_-substituted dipropionic acid, were administered to rats. The following CsA solutions were administered intraduodenally: (1) CsA/levan/BC as a control (2 mg·kg^−1^ of CsA, levan (0.5 *w*/*v*%), and BC (0.5 *w*/*v*%)), (2) CsA/levan/BC with 5 mg·kg^−1^ of Pep-4, and (3) CsA/levan/BC with 10 mg·kg^−1^ of Pep-4.

**Figure 3:**
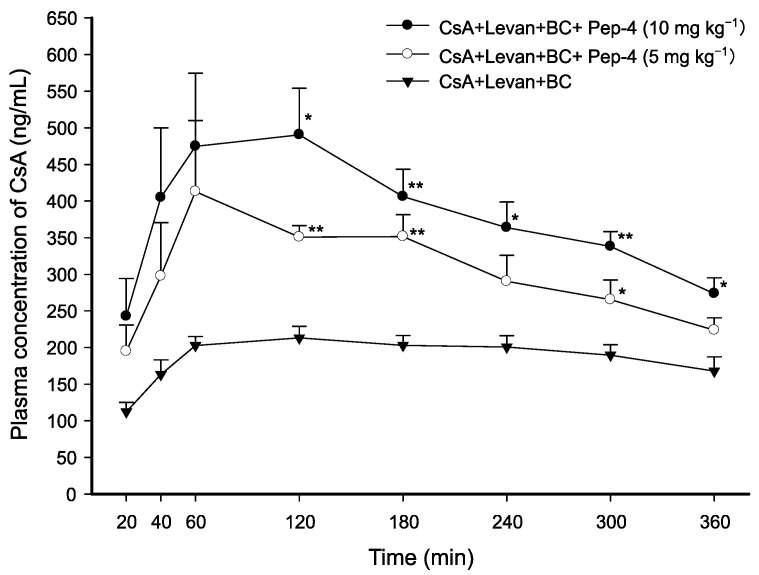
Temporal profiles of CsA concentration in the plasma after intraduodenal administration of CsA solutions with varying concentrations of Pep-4. The dose of CsA was 2 mg·kg^−1^. Data are presented as mean ± SEM of 3–4 rats. Significant differences are indicated with * *p* < 0.05 and ** *p* < 0.01 compared with CsA/levan/BC control solution.

The administration of the solution containing CsA, levan, and BC with 10 mg·kg^−1^ of Pep-4 significantly increased the plasma concentrations of CsA. The increases were observed at different time points: 2.30-fold (*p* < 0.05) at 120 min, 2.00-fold (*p* < 0.01) at 180 min, 1.81-fold (*p* < 0.05) at 240 min, 1.78-fold (*p* < 0.01) at 300 min, and 1.63-fold (*p* < 0.05) at 360 min compared to the control group, which received only the CsA/levan/BC solution (Figure 3). When the CsA solution with levan and BC was administered along with a reduced concentration of Pep-4 (5 mg·kg^−1^), the plasma concentrations of CsA were increased by only 1.65-fold (*p* < 0.01) at 120 min, 1.73-fold (*p* < 0.01) at 180 min, and 1.40-fold (*p* < 0.05) at 300 min. Additionally, the solution containing CsA, levan, and BC with 10 mg·kg^−1^ of Pep-4 significantly enhanced the AUC_0–360min_ and C_max_ by 2.03-fold (*p* < 0.05) and 2.37-fold (*p* < 0.01), respectively. In contrast, the solution with 5 mg·kg^−1^ of Pep-4 only increased the AUC_0–360min_ and C_max_ by 1.62-fold (*p* < 0.01) and 2.18-fold (*p* < 0.01), respectively, when compared to the CsA control solution (Table 2).

These results suggest that the cysteine-substitution in FCIGRL affects its ability to enhance permeation. Furthermore, the intraduodenal absorption of CsA increased proportionally with the concentration of Pep-4 in the presence of levan and BC.

## 4. Discussion

This study used four synthesized peptides modified from FCIGRL as absorption enhancers and evaluated the effectiveness of these modified peptides on intestinal absorption of CsA. Previous studies have reported that ZOT and its fragment, FCIGRL, can significantly increase membrane permeation of CsA in both in vitro and in vivo studies. ZOT at a concentration of 4 μg·mL^−1^ improved the apparent permeability coefficient (*P_app_*) of CsA by 1.21-fold compared to the control in Caco-2 cells [12]. FCIGRL at a dose of 10 mg·kg^−1^ enhanced AUC_0–120min_ by 2.14-fold and the C_max_ of CsA by 2.56-fold after administration of CsA with PIs and BC intraduodenally into rats [17]. Additionally, FCIGRL-NH_2_, which corresponded to Pep-1 in this study, was known to increase the AUC and C_max_ of mannitol by 3.63-fold and 2.68-fold, respectively, after intranasal administration to rats, while FCIGRL increased the AUC and C_max_ by 1.48-fold and 1.25-fold, respectively, compared to the control in the mannitol study [18]. Moreover, FCIGRL-NH_2_ produced a 2.02-fold increase in the AUC of atenolol in the presence of levan compared to the control of atenolol alone [19].

Therefore, it was expected that Pep-1 in the presence of levan and BC could also increase the absorption of CsA. However, in this study, Pep-1 only resulted in a 1.50-fold increase (*p* < 0.05) in the C_max_ and no difference in AUC_0–360min_ compared to the control. Although these results are not directly comparable to the previous CsA study using FCIGRL with different peptide stabilizers and CsA doses, it appeared that the absorption-enhancing efficacy of CsA by Pep-1 was lower than that by FCIGRL. However, the lower efficacy of Pep-1 than FCIGRL can be explained by differences in stabilizers for peptides according to the results of Pep-2 and Pep-3.

Pep-2 and Pep-3 differ only in the amidation of the C-terminus, like FCIGRL and Pep-1. Pep-3 resulted in higher enhancement of AUC_0–360min_ and C_max_ of CsA with higher absorption of CsA compared to Pep-2. These results are consistent with previous papers showing that amidation of the C-terminus can affect stabilization of the peptide, further increasing the absorption promotion effect [18,19]. In addition, Pep-2 and Pep-3, in that order, showed higher absorption-enhancing efficacy for intestinal absorption of CsA compared with Pep-1. These results indicated that cysteine dimerization contributed to the stabilization of the peptides apart from amidation of the C-terminus, consistent with previous papers showing that homo-dimerization improved the stability of peptide complex [23,24].

FCIGRL interacts with proteinase-activated receptor-2 (Par-2), impacting the structure and function of tight junctions and increasing membrane permeability [25,26,27]. Since FCIGRL-modified peptides share three amino acid residues “-GRL” in the carboxyl-terminus with the putative receptor binding site of ZOT [28], results in this study suggest that permeation-enhancing properties of Pep-2 and Pep-3 might also be induced by interaction with the Par-2 receptor. Studies are underway to clarify the specific role of the Par-2 receptor in the biological activity of FCIGRL-modified peptides.

In Pep-4, the cysteine of Pep-1 was replaced with a relatively lipophilic N_3_-substituted dipropionic acid. It was assumed that the peptide’s lipophilicity could positively affect its interaction with the Par-2 receptor. Results showed that Pep-4 also enhanced the intestinal absorption of CsA, similar to Pep-3. It increased the AUC_0–360min_ and C_max_ of CsA in proportion to its concentration when levan and BC were present. These findings indicate that Pep-2, Pep-3, and Pep-4 could be used as intestinal absorption enhancers for CsA in the presence of levan and BC.

## 5. Conclusions

This study assessed the effectiveness of amino acid-modified peptides, derived from FCIGRL-OH, as improved absorption enhancers for the intestinal permeation of CsA in rats. The findings indicate that modifications to the chemically reactive sites in FCIGRL-OH, specifically dimerizing the cysteine residue at position 2, substituting it with an N3-substituted dipropionic acid, or capping the C-terminus with an amide group, play a critical role in improving both the stability of these peptides and their absorption-enhancing effects. As a result, the study identifies Pep-2, Pep-3, and Pep-4 as promising absorption enhancers for CsA, particularly when used in combination with levan and BC. This approach represents a valuable strategy for increasing the intestinal permeation and bioavailability of CsA.

## Figures and Tables

**Table 1:** Structures of modified peptides derived from FCIGRL.

Peptide	F-X-IGRL		Dimerization
	X	C-Terminus	
Pep-1	Cysteine	-CONH_2_	-
Pep-2	Cysteine	-COOH	Cysteine homo-dimer
Pep-3	Cysteine	-CONH_2_	Cysteine homo-dimer
Pep-4	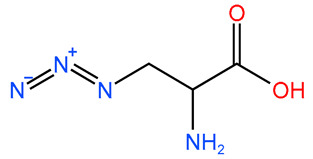	-CONH_2_	-

**Table 2:** Pharmacokinetic parameters after intraduodenal administration of CsA (2 mg·kg^−1^) with each FCIGRL-modified peptide, levan (0.5 *w*/*v*%) and BC (0.5 *w*/*v*%).

CsA + Levan + BC	AUC_0–360min_ (min·ng·mL^−1^)	C_max_ (ng·mL^−1^)	T_max_ (min)	T_1/2_ (min)	V_d_/F (L·kg^−1^)	Cl/F (mL·min^−1^·kg^−1^)
+ Pep-4 (10 mg·kg^−1^)	136,063.27 ± 16,010.40 * (2.03-fold)	511.91 ± 76.37 * (2.37-fold)	90.00 ± 17.32 (0.86-fold)	280.61 ± 6.77 (0.96-fold)	3.37 ± 0.31 ** (0.54-fold)	8.34 ± 0.81 * (0.56-fold)
+ Pep-4 (5 mg·kg^−1^)	108,567.23 ± 8691.20 ** (1.62-fold)	470.70 ± 41.30 ** (2.18-fold)	100.00 ± 40.00 (0.95-fold)	262.97 ± 36.95 (0.90-fold)	3.90 ± 0.15 * (0.63-fold)	10.63 ± 1.30 (0.71-fold)
+ Pep-3 (10 mg·kg^−1^)	134,627.65 ± 5928.82 ** (2.01-fold)	529.73 ± 29.55 ** (2.46-fold)	120.00 ± 0.00 (1.14-fold)	289.85 ± 37.15 (1.00-fold)	3.59 ± 0.34 * (0.58-fold)	8.66 ± 0.34 * (0.58-fold)
+ Pep-3 (5 mg·kg^−1^)	88,473.93 ± 15,317.75 (1.32-fold)	384.55 ± 82.62 (1.78-fold)	90.00 ± 17.32 (0.86-fold)	299.42 ± 9.85 (1.03-fold)	5.47 ± 0.99 (0.88-fold)	12.73 ± 2.44 (0.85-fold)
+ Pep-2 (10 mg·kg^−1^)	116,743.61 ± 13,310.67 ** (1.74-fold)	500.02 ± 6.78 ** (2.32-fold)	120.00 ± 0.00 (1.14-fold)	264.41 ± 5.44 (0.91-fold)	4.08 ± 0.39 (0.66-fold)	10.72 ± 1.23 (0.71-fold)
+ Pep-1 (10 mg·kg^−1^)	91,231.24 ± 9560.07 (1.36-fold)	323.02 ± 29.21 * (1.50-fold)	140.00 ± 20.00 (1.33-fold)	263.44 ± 42.10 (0.90-fold)	4.95 ± 0.44 (0.80-fold)	13.91 ± 2.83 (0.93-fold)
CsA + Levan + BC (control)	67,055.95 ± 4429.74	215.69 ± 14.38	105.00 ± 28.72	291.24 ± 24.81	6.21 ± 0.66	15.01 ± 1.71

Data are presented as mean ± SEM of 3–4 rats. Values in the bracket indicate enhancement ratio of each pharmacokinetic parameter compared to CsA including levan and BC (*, *p* < 0.05; **, *p* < 0.01). Abbreviation: CsA, Cyclosporin A; BC, Benzalkonium chloride; AUC, Area under the curve; C_max_, Maximum plasma concentration; T_max_, Time to reach the maximum concentration; V_d_, Volume of distribution; Cl, Clearance; F, Bioavailability.

## Data Availability

Data presented in this study are contained within the article. Further inquiries can be directed to the corresponding author.
